# The role of *Klebsiella* populations in preterm infants

**DOI:** 10.1042/BST20200325

**Published:** 2023-04-26

**Authors:** Anne L. McCartney, Lesley Hoyles

**Affiliations:** Department of Biosciences, School of Science & Technology, Nottingham Trent University, Nottingham, U.K.

**Keywords:** *Klebsiella oxytoca* complex, *Klebsiella pneumoniae*, microbial ecology, microbiota, necrotizing enterocolitis, sepsis

## Abstract

The preterm infant microbiota is dominated by *Enterobacteriaceae* (*Escherichia*, *Klebsiella* or *Enterobacter* spp.), *Enterococcus* and *Staphylococcus* spp. Recent work has demonstrated the development of this microbiota is predictable and driven by simple microbe–microbe interactions. Because of their systemic immaturity, including an underdeveloped immune system, preterm infants are susceptible to a range of infections. Numerous retrospective studies have examined the association of the preterm gut microbiota with diseases such as necrotizing enterocolitis (NEC), early-onset sepsis and late-onset sepsis. To date, no single bacterium has been associated with infection in these infants, but a *Klebsiella*/*Enterococcus*-dominated faecal microbiota is associated with an increased risk of developing NEC. Staphylococci aid and enterococci inhibit establishment/maintenance of gastrointestinal *Klebsiella* populations in preterm infants, though the mechanisms underlying these interactions are poorly understood. *Klebsiella* spp. recovered from healthy and sick preterm infants display similar antimicrobial resistance and virulence profiles, giving no clues as to why some infants develop potentially life-threatening diseases while others do not. The identification of cytotoxin-producing *Klebsiella oxytoca sensu lato* in the gut microbiota of some preterm infants has led to the suggestion that these bacteria may contribute to NEC in a subset of neonates. This mini review highlights current knowledge on *Klebsiella* spp. contributing to the preterm gut microbiota and provides insights into areas of research that warrant further attention.

## The genus *Klebsiella*

**Figure 1.**
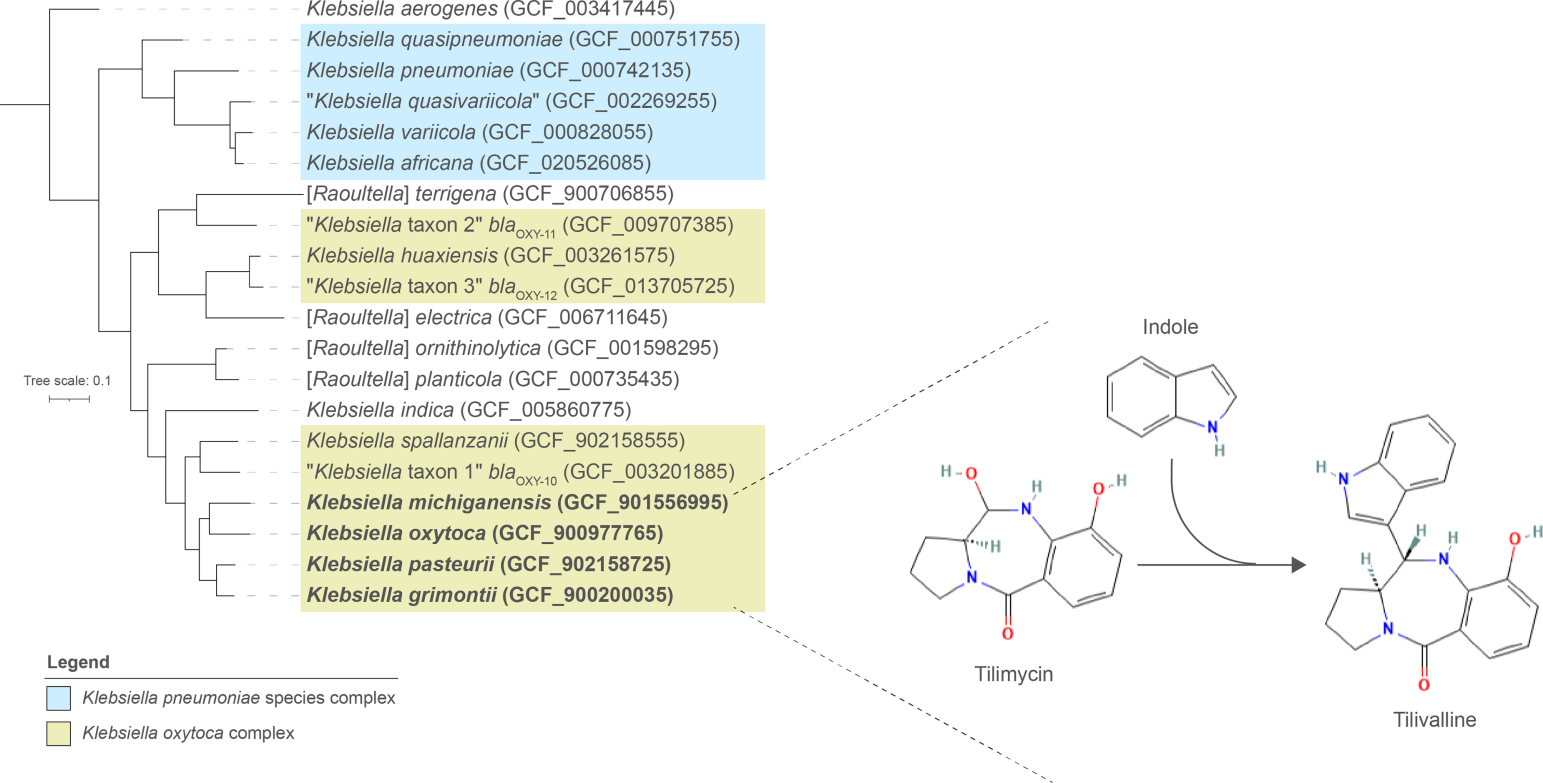
The *K. pneumoniae* species complex and the *K. oxytoca* complex. The phylogenetic tree shows the genus *Raoultella* is intermixed with the genus *Klebsiella*. Strains of some species of the *K. oxytoca* complex (shown in bold text) can produce the cytotoxic pyrrolobenzodiazepine metabolite tilimycin (TM), which spontaneously reacts with indole to form tilivalline (TV) (discussed later in the text); TM and TV are causative agents of antibiotic-associated haemorrhagic colitis [69]. The tree, rooted at *K. aerogenes*, was created from an alignment of 338 core protein sequences using PhyloPhlAn3 [80] and visualized using iToL v6 [81]. Scale bar, average number of amino acid substitutions per position.

As of February 2023, the genus *Klebsiella* (family *Enterobacteriaceae*) encompassed 13 species of bacteria with validly published names and one species with a non-valid name. *Raoultella* spp. are also considered part of the genus *Klebsiella* (Figure 1) [1,2]. *Klebsiella pneumoniae*, *Klebsiella oxytoca* and *Klebsiella aerogenes* (formerly *Enterobacter aerogenes* [3]) have received most attention from a clinical perspective, with *K. pneumoniae* responsible for up to 15% of healthcare-associated infections and increasing levels of antimicrobial resistance being reported for all species [4–7]. *Klebsiella* spp. are found in the environment, and contribute to the commensal gut microbiota of humans and animals. Gut colonization with *Klebsiella* spp. contributes to extraintestinal infections in the immunocompromised and clinically vulnerable [7]. *K. oxytoca* and *K. aerogenes*, and to a lesser extent *Raoultella* spp., represent emerging pathogens [4–6].

In recent years, the adoption of whole-genome sequencing in taxonomic, clinical and epidemiological studies has led to an increased understanding of the genetic diversity of *Klebsiella* spp. (Figure 1). *K. pneumoniae* has a diverse population structure, representing a complex of five species [*K. pneumoniae*, *K. quasipneumoniae* (subsp. *quasipneumoniae* and *similipneumoniae*), ‘*K. quasivariicola*', *K. africana*, *K. variicola* (subsp. *tropica* and *variicola*)] [7]. The *K. oxytoca* complex comprises several distinct phylogroups (Ko) defined based on differences in *bla*_OXY_ sequences [*K. michiganensis* (Ko1, Ko5), *K. oxytoca* (Ko2), *K. spallanzanii* (Ko3), *K. pasteurii* (Ko4), *K. grimontii* (Ko6), *K. huaxiensis* (Ko8) and three unnamed novel species] [4]. The contribution *of K. oxytoca* to human clinical infections has likely been over-estimated, with *K. michiganensis* more prevalent based on retrospective analyses of publicly available genome data [4,8].

## Preterm infants

Preterm describes infants born prematurely [i.e. <37 weeks gestational age (GA)], and they are often of low birth weight (LBW; <2.5 kg). Approximately 11% of global live births are preterm, and account for a third of all neonate deaths [9]. Intestinal colonization of preterm infants occurs in neonatal intensive care units (NICUs), with empiric antibiotics administered to most, but not all, preterm infants in the first days of life to cover possible early-onset infection from birth contributing to the development of a gut microbiota dominated by *Enterobacteriaceae*, *Enterococcus* and *Staphylococcus* spp. [10,11]. Between 1 and 10% of preterm infants harbour *Klebsiella* spp. in their faecal microbiota [12], but this proportion can be much higher depending on geographical location [10,13]. Colonization with these, and other opportunistic pathogens, along with an unstable microbiome and systemic developmental immaturity (especially with respect to immune and gastrointestinal functions) contribute to nosocomial infections such as early-onset sepsis (EOS; <72 h after birth), late-onset sepsis (LOS; ≥72 h after birth), necrotizing enterocolitis (NEC; infection and inflammation of the small and large intestines that can progress to necrosis, sepsis and death) in this patient group [12]. Infants born at <32 weeks GA and of very low birth weight (VLBW; <1.5 kg) are particularly susceptible to infection. A recent study in China (Sina-Northern Neonatal Network) looked at the incidence of LOS in 6639 VLBW infants admitted to 35 different NICUs over a 3-year period. From the LOS cases recorded, 456/1511 (30%) positive cultures were obtained from blood: among these *K. pneumoniae* was the bacterium most often associated with LOS (147/456, 32%) [14]. However, it is clear from other retrospective studies that the proportion of preterm infections that *K. pneumoniae* contributes to (between 9 and 76%; summarizing data for sepsis, which includes EOS and LOS; Supplementary Table S1) depends on geographical location.

In this review, we aim to summarize the current understanding of *Klebsiella* spp. in relation to preterm infants and other information regarding *Klebsiella* that may be relevant to these neonates, and to highlight the need for further research to unravel the role(s) of *Klebsiella* spp. in this patient population.

## Retrospective studies

What do we know or understand about *Klebsiella* spp. and their interactions with preterm infants? The answer is very little. This is largely because there has, until recently, been very little interest in or focus on these bacteria in this patient group. Curation of a PubMed and Web Of Science search for original research articles published in the last 5–10 years using the terms ‘*Klebsiella*' and ‘preterm infants' reveals many available scientific articles are retrospective/observational studies (Supplementary Table S1) [15–50]. Most of these studies do not focus on *Klebsiella* spp. specifically, instead they include information on a range of bacteria, predominantly ESKAPE pathogens (*Enterococcus faecium*, *Staphylococcus aureus*, *Klebsiella pneumoniae*, *Acinetobacter baumannii*, *Pseudomonas aeruginosa*, *Enterobacter* spp.), that are known to be associated with nosocomial infections, antimicrobial resistance and/or preterm infants and different neonatal health outcomes. The overriding message is that *Klebsiella* spp., along with a range of other opportunistic pathogens, contribute to disease in preterm populations globally. No single bacterium has been linked to preterm-associated infections. However, faecal microbiotas dominated by *Klebsiella*, or *Klebsiella* and *Enterococcus* spp. have been described as two of six preterm gut community types, with the latter type more commonly associated with a NEC diagnosis [10].

While retrospective studies are relatively inexpensive to run and allow consideration of multiple outcomes, with outcome data already available, they are not without disadvantages. They do not provide a true understanding of the relationship between preterm infants and different bacterial groups, including *Klebsiella* spp. It is often not possible to link time of exposure of a preterm infant to a specific bacterium to subsequent infection, and high numbers of patients are required to detect rare associations. Differences in definitions of disease state and inclusion criteria also influence study outcomes [10]. Significant biases may be introduced to retrospective studies based on culture medium and/or cultivation environment selected: many of the documented studies have relied on clinical microbiological data generated using a broad range of microbiological cultivation techniques known to pick up easily cultivated microbes or predominant clinical isolates. To investigate the association/role of *Klebsiella* spp. with preterm infant health outcomes, study design should include specific focus on *Klebsiella*, whether that be using appropriate cultivation methods [*Klebsiella*-specific agar(s) and/or culture conditions] or molecular tools and analyses focused on *Klebsiella*-specific genetic targets. In addition, such research should involve as wide a range of preterm infants as possible and a range of sample types, not simply those preterm infants with specific clinical outcomes/conditions which necessitate sampling (e.g. blood, sputum, stool) for clinical microbiology aimed at informing subsequent therapeutic regimes and identifying/monitoring outbreaks.

## *Klebsiella* spp. and the preterm infant gut microbiota

The younger their GA, the more underdeveloped are the organs and immune system of preterm infants. However, as the immune system of full-term infants is also immature at birth, the immaturity of preterm infants’ other systems (including intestinal motility and secretions, digestion, absorption, mucosal surfaces, barrier function and circulatory regulation) also contributes to their increased susceptibility to neonatal infections [51].

Increased exposure to maternal cytokines (e.g. TNF-α, IL-1, IL-6 and IL-8) can have immunomodulatory effects on preterm infants [9,52]. It has been proposed that these may contribute to the protection of VLBW infants against infection and the acceleration of lung maturation [53]. Although the preterm immune system is underdeveloped, the gut of these neonates is highly immunoreactive and has an exaggerated pro-inflammatory response in NEC [51,54]. The lipopolysaccharide receptor TLR-4 contributes to normal development of the small intestine, but in preterm infants its expression is increased compared with full term infants; its *in vivo* activity can be inhibited by amniotic fluid and breast milk, and reduced by sodium butyrate [54]. TLR-4 of preterm infants is activated *in utero* and by Gram-negative members of the preterm gut microbiota (*Enterobacteriaceae*, including *Klebsiella* spp.): its activation leads to recruitment of pro-inflammatory T helper 17 cells and release of pro-inflammatory cytokines (IL-17, IL-22), eventually leading to erythrocyte death, mucosal injury and translocation of bacteria to the microvasculature underlying the intestinal epithelium [54]. TLR-4 activation also mediates loss of enteric glia, resulting in impaired intestinal motility and hyperinflammation [54]. The cytokine IL-8, which mediates migration and activation of neutrophils to sites of inflammation, is produced by intestinal epithelial cells, and can contribute to necrosis in the preterm gut [51].

The differential exposure of preterm and full-term infants to antibiotic therapy, microbiological and environmental components, immune status and hospital stay influence neonatal microbiome development in these infant groups [55]. Several recent studies have investigated the gut microbiota of preterm infants, in some cases focusing on specific neonatal clinical outcomes (Supplementary Table S1) and in other cases considering diet [synbiotics, prebiotics, probiotics or different milks (breast milk, fortified breast milk, formula milks)] or therapeutic regimes targeting improved clinical outcomes [56–61].

Ho et al. [62] used 16S rRNA gene amplicon sequencing to characterize the faecal microbiota of VLBW infants (*n* = 45; GA 28 ± 2 weeks; birth weight 1126 ± 208 g) in a NICU (South Florida and Tampa General Hospital, U.S.A.) during the first month of life, sampling each infant at ≤2 weeks, 3 weeks and 4 weeks postnatally. Proteobacteria (46% of total microbiota, mostly Gammaproteobacteria) and Firmicutes (41%, mostly Bacilli) predominated initial samples (≤2 weeks). Actinobacteria and Bacteroidetes were minor components of the gut microbiota of this study cohort. Relative abundance of Gammaproteobacteria increased throughout the study: from 42.5% (≤2 weeks) to 69.7% (3 weeks) to 75.5% (1 month). However, there was wide interindividual variation in carriage of Gammaproteobacteria in neonates initially (0–90%), with two clusters defined based on Gammaproteobacteria abundance (cluster 1, 2.1 ± 5.9%, *n* = 20 infants; cluster 2, 79.2 ± 21.6, *n* = 24 infants). Cluster 1 infants were more likely to have been delivered by Caesarean section and had lower birth weight than cluster 2 infants, and seemingly had delayed establishment of Gammaproteobacteria (in terms of abundance) compared with cluster 2. *Klebsiella* was the most abundant genus observed in the gut microbiota of cluster 2 infants, with a single amplicon sequence variant dominating. A subsequent publication from the same authors examined the relationship between faecal Gammaproteobacteria and faecal calprotectin (a biomarker for mucosal inflammation) in this study cohort [63]. While they again discussed two subgroups of the cohort (clusters 1 and 2), their distinction of clusters was different — based on *Klebsiella* [63] rather than Gammaproteobacteria abundance [62] (with three infants moving from cluster 2 [62] to cluster 1 [63]). Faecal calprotectin levels were significantly correlated with *Klebsiella* abundance (*r* = 0.207, *P *< 0.05), with cluster 1 having lower faecal calprotectin than cluster 2 (148 vs 226 µg/g stool at ≤2 weeks postnatally, *P *< 0.05). Correlation does not equate with causation or mechanistic understanding of associations. Therefore, the question arises as to whether mucosal inflammation influences *Klebsiella* colonization, or *vice versa*.

Two recent studies characterized the faecal microbiota of preterm infants during the first 4–6 weeks of life to determine early microbiota development [13,64]. Heida et al. [64] demonstrated an initial abundance of *Staphylococcus* spp., which transitioned to an *Enterobacteriaceae*-dominated microbiota during the first month postnatally in a weight-associated manner. Delivery mode was shown to influence the initial gut microbiota, with *Escherichia* and *Bacteroides* spp. more common in the faeces of vaginally delivered infants. *Klebsiella* spp. were a common/normal member of the developing neonatal microbiota [64]. Rao et al. [13] collected faecal samples from 178 preterm infants fortnightly during the first 6 weeks of life (at 1, 14, 28 and 42 days), together with near daily sampling from 13 of the cohort. Similar to other studies [10,64], initial dominance of the faecal microbiota with staphylococci was seen, with subsequent transition to a *Klebsiella-*, *Enterococcus-* or *Escherichia*-dominated microbiota with age. By using a scalable multi-kingdom absolute abundance quantification method, ecological modelling and *in vitro*/*in vivo* validations, Rao et al. [13] were able to demonstrate the predictability of assemblage of the preterm microbiota, driven by simple microbe–microbe interactions. Establishment of *Klebsiella* spp. in the gut was facilitated by *Staphylococcus* spp., with klebsiella suppressing growth of staphylococci. *Klebsiella* spp. were inhibited by enterococci. The factors produced by staphylococci and enterococci that influence the growth of *Klebsiella* spp. warrant further attention. Rao et al. [13] also demonstrated that reliance on relative abundance data in microbiota profiling studies can skew findings, potentially masking microbial dynamics (especially *Klebsiella* and *Escherichia* ‘blooms’ commonly associated with pre-disease states in preterm infants). In addition, they highlighted that correlation analysis in no way predicted the dynamics or ecological processes underlying development of the preterm gut microbiota. These findings have implications for the wider microbiome research community.

Seki et al. [65] examined multiple data points of extremely preterm neonates (<28 weeks GA) related to the gut microbiota–immune–brain axis. This observational study included 53 infants (*n* = 15 diagnosed with severe brain injury; *n* = 38 had no/mild brain injury), with magnetic resonance imaging scans (brain development), peripheral blood samples (immune markers) and regular stool samples (gut microbiota; day 3, day 7 and fortnightly samples week 2 to week 12 after birth) analyzed. *Klebsiella* spp. contributed to the gut microbiota of extremely preterm neonates, together with nine other genera described as prevalent (found at ≥10^5^ cells in at least 1/5 faecal samples). *Klebsiella* spp. were less abundant in early stool samples (days 3 and 7) of neonates with severe brain injury, but more abundant (1.7 times higher) at 4 and 6 weeks of age compared with neonates with no/mild brain injury. Elevated *Klebsiella* abundance was associated with white-matter injury, as were a number of immune-related components in peripheral blood (C-reactive protein, specific T-cell populations, and several cytokines/chemokines) [65]. Mechanistic studies are required to unravel the relevance of these associations with preterm infant health.

Olm et al. [66] investigated the gut microbiota associated with NEC, using shotgun metagenomic analysis of biobanked faecal samples of NEC patients and matched controls. UniFrac analysis of microbiota data did not show distinct clustering of NEC and control neonates. However, *K. pneumoniae* was enriched in samples from infants subsequently diagnosed with NEC, detected in 52% of pre-NEC samples compared with 23% of control samples. When bacterial replication rates (iRep values) were considered rather than relative abundances from metagenomic data, significantly higher replication rates (total microbiota) were seen in pre-NEC samples compared with controls. Machine learning-informed analyses of metagenomic data identified four aspects of the preterm gut microbiome that differed between pre-NEC and control samples: iRep value (total microbiota), *Klebsiella* spp., secondary metabolite clusters and fimbriae that could elicit a TLR4-mediated pro-inflammatory response [66]. Similar to Rao et al. [13], these authors highlighted reliance of relative abundance data (without consideration of measures such as iRep) could lead to misleading interpretation of study findings.

## Diet, *Klebsiella* spp. and the preterm infant microbiota

Diet-related differences in the faecal microbiota of preterm infants have been observed. Pärnänen et al. [67] found several ESKAPE organisms (including *K. pneumoniae*), as well as *K. oxytoca* and *Clostridioides difficile*, enriched in the faecal microbiota of formula-fed infants compared with breast-fed or fortified human-milk-fed infants. A study focused on feeding intolerance and the gut microbiota of preterm infants suggested that the relative diversity of the gut microbiota significantly decreased in association with a diagnosis of feeding intolerance [68]. A *Klebsiella*-dominant faecal microbiota was observed for the feeding-intolerance group when this intolerance was diagnosed (stool collected within 24 h of diagnosis), although this may partially reflect the reduced diversity of the microbiota (i.e. loss of species richness rather than an increased abundance of *Klebsiella* spp.) [68]. However, it should be noted that *Klebsiella* spp. are lactose-fermenting bacteria — increased abundance of these bacteria in feeding-intolerant preterm infants may be due to microbial catabolism of lactose included in some enteral feeds.

## Virulence of *Klebsiella* spp. and cytotoxicity

We investigated *Klebsiella* populations associated with the faecal microbiota in a U.K. preterm cohort (*n* = 109, <37 weeks GA) [12]. Microbiota profiling (amplicon-based) demonstrated 38.5% of infants harboured *Enterobacteriaceae* in their first available stool sample after birth. Cultivation work recovered *Enterobacteriaceae* from 42.2% of the same faecal samples. Multiple species of *Enterobacteriaceae* were harboured by some infants, while others appeared to have a single predominant species (based on colony morphology and biochemical data from distinctive colony types) [12]. Most infants harbouring lactose-fermenting *Enterobacteriaceae* (i.e. *Klebsiella*, *Escherichia* and *Enterobacter* spp.) were healthy preterm infants (*n* = 23), three had a NEC diagnosis during their stay in NICU, there were eight cases of suspected sepsis, one infant had an eye infection and one had an operation (gastroschisis). No common *Klebsiella* strains were found among the infants in the cohort, with virulence- and antimicrobial-associated differences observed among genome sequences of isolates from each infant, even when they shared sequence, capsule and/or O-antigen types. Eight *K. pneumoniae*, three *K. grimontii*, two *K. michiganensis* and one *K. quasipneumoniae* strains were isolated. Faecal *Klebsiella* isolates (from both healthy and sick infants) were able to reside, persist and potentially replicate in macrophages, suggesting they could all evade the host immune system and had the potential to cause opportunistic infections [12]. Preterm infants receive a range of iron supplements (blood transfusions, parenteral feeding, oral) while in NICUs during the first weeks of life. All *Klebsiella* strains recovered from healthy and sick infants produced siderophores (iron scavengers) *in vitro*, demonstrating no difference in the colonization or virulence potential of these bacteria [12]. Consequently, much research is required to determine the interactions between neonate and *Klebsiella* spp. that lead to disease in some preterm infants.

Tilimycin (TM) and tilivalline (TV) are cytotoxic pyrrolobenzodiazepine metabolites (Figure 1). TM, a DNA-damaging agent, is encoded by a biosynthetic gene cluster (BGC) in the genomes of some strains of species belonging to the *K. oxytoca* complex (specifically *K. oxytoca*, *K. michiganensis*, *K. grimontii* and *K. pasteurii*); TM spontaneously reacts with indole to form TV, which targets tubulin and disrupts the spindle apparatus of eukaryotic cells [8,69]. These metabolites are causative agents of antibiotic-associated haemorrhagic colitis (AAHC; diffuse mucosal oedema, haemorrhagic erosions, bloody diarrhoea), with disease caused by the overgrowth of cytotoxin-producing strains secondary to the use of antibiotics [70,71]. A recent study in mice has demonstrated the DNA-alkylating metabolite TM causes the accumulation of mutations in cycling intestinal stem cells within weeks of a single *K. oxytoca* overgrowth, driving somatic changes that could hypothetically contribute to disease susceptibility in some preterm infants who are subject to transient ‘blooms’ of TM-producing bacteria in their gut [72]. We have identified strains of *K. grimontii* in the faecal microbiota of preterm infants that encode the BGC, along with metagenome-assembled genomes of *K. michiganensis* and *K. oxytoca* recovered from faecal samples of preterm infants [8,12]. Around the same time as our study [8], using a *m*-hydroxybenzoate agar that selects for *K. oxytoca*-related bacteria over other *Klebsiella* spp., Paveglio et al. [73] recovered strains from preterm infants (<32 weeks GA) with NEC that could produce both TM and TV (confirmed by mass spectrometry analysis and apoptosis assays). They identified the strains as cytotoxin-producing *K. oxytoca* based on 16S rRNA, *pehX* (a marker for *K. oxytoca sensu lato*) and *npsAB* (non-ribosomal peptide synthetase genes A and B essential for the synthesis of TM) gene sequence analyses, but they were in fact strains of *K. grimontii* and *K. pasteurii* based on reanalysis of their multilocus sequence data [8]. They then used frozen stool samples from NEC and non-NEC matched controls to isolate *K. oxytoca* from the gut microbiota. Cytotoxin-producing *K. oxytoca* was isolated from 6/10 and 4/5 of the NEC and non-NEC infants, respectively, with 4/10 and 1/5 harbouring cytotoxin-negative strains of the bacterium. Interestingly, the relative abundance of *K. oxytoca* from 16S rRNA gene-based amplicon profiling from these infants’ faecal samples differentiated NEC infants harbouring *nspAB*-positive *K. oxytoca* (high relative abundance of *K. oxytoca*) from all other infants (NEC patients with toxin-negative *K. oxytoca* and control infants, low abundance) [73]. Furthermore, the presentation of NEC symptoms was different for patients in accordance with the timing of high abundance of *K. oxytoca* relative to NEC onset (high *K. oxytoca* abundance ‘prior to’ versus ‘near or shortly after’ onset). This may reflect differences in antibiotic treatment regimens (course duration and proximity to NEC onset) used for these cases given the known association of AAHC with antibiotic administration.

As mentioned above, preterm infants are given empiric antibiotics in the first days to weeks of life, and — similar to AAHC — blood in the stool and intestinal necrosis are hallmarks of NEC. The finding of cytotoxin-producing strains of the *K. oxytoca* complex in the faeces of preterm infants has led to the suggestion that these bacteria could contribute to NEC in a proportion of preterm infants [8,73]. Healthy infants (weeks 0–8 of life) in an Austrian cohort had a carriage rate of *K. oxytoca sensu lato* of 49–73%, with PCR-based assays leading to higher rates of detection than cultivation work; ∼50% of recovered isolates were cytotoxic, though it was noted that not all *npsAB*-positive strains produced cytotoxin as assessed by MTT and chemical analyses [74]. A recent study examined a published metagenomic dataset [75] derived from 829 faecal samples from 571 full-term infants born in the U.K. for the presence of *K. oxytoca* complex bacteria [72]. At days 7 and 21 of life, 76/504 (15%) and 74/325 (23%) samples, respectively, harboured toxigenic *Klebsiella* spp. Of the complex-positive samples, 46/76 encoded the minimal *til* locus (BGC) at day 7 (in order of species prevalence: *K. grimontii*, *n* = 34/37; *K. michiganensis*, *n* = 4/25; *K. oxytoca*, *n* = 6/11; *K. pasteurii*, *n* = 2/3), with prevalence of each BGC-positive species increasing between days 7 and 21 of life (from 9.1 to 12.6%, *n* = 41/74). Across all BGC-positive samples, the relative abundance of BGC-encoding bacteria was high (range 0.92–94.1%, median 12.6%) [72]. No robust data are available for the gut carriage of *K. oxytoca sensu lato* (cytotoxic or otherwise) by preterm infants. Therefore, it is clear the association of cytotoxin-producing *Klebsiella* spp. with diseases affecting preterm infants is complex and warrants further study. Adoption of the real-time PCR assay for *npsAB* genes [76], with a sensitivity of 15 cfu/ml of sample, for the detection of potential cytotoxin*-*producing bacteria in the faeces of preterm infants would allow us to better define the relationship between these pathogens and the presentation of NEC in this cohort. In addition, it is clear that refined molecular-based identification [whole genome (15), shotgun metagenomic [12] or *rpoB* gene [77] sequencing] should be adopted to accurately identify members of the *K. oxytoca* complex in the gut microbiota of preterm infants and to correctly identify clinically relevant isolates.

TM can inhibit the growth of *Lactobacillus*, *Bacteroides*, *Fusobacterium*, *Proteus* and *Bifidobacterium* spp., members of the commensal gut microbiota [69,78]. In the U.K., probiotic interventions in neonates most frequently involve feeding *Bifidobacterium* and *Lactobacillus*-containing multi-strain preparations [57]. Whether TM inhibits and/or influences intestinal colonization with probiotic bacteria remains to be studied. It has been hypothesized that TM confers cytotoxin-producing strains with a competitive advantage over other gut bacteria when in the presence of an appropriate carbon source (e.g. glucose). Support for this suggestion comes from a study done with *in vitro* systems inoculated with human faeces that showed TM (1–170 µM) exerted broad-spectrum activity against a range of Gram-positive and Gram-negative gut bacteria [79]. In mice TM caused reductions in the species richness and evenness of the murine gut microbiota, driving compositional changes [79]. In addition, and of great concern with respect to the global and preterm burden of antimicrobial resistance in ESKAPE pathogens, TM directly contributed to *de novo* mutations in the genomes of *Escherichia coli* and *K. pneumoniae* strains that led to their resistance to rifampicin and nalidixic acid; a strain *of Pseudomonas aeruginosa* also acquired a resistance determinant associated with rifampicin upon exposure to TM [79]. No antibacterial effect has been demonstrated for TV to date, either in pure or mixed culture or the murine microbiota [79].

## Perspectives

- *Klebsiella* spp. are often part of the normal gut microbiota of preterm infants, and sometimes contribute to infectious diseases affecting this patient population.
- The current literature is dominated by retrospective studies. Shotgun metagenomic and cultivation-based studies of the preterm faecal microbiota have shown *Klebsiella* spp. contributing to infections in preterm infants are phenotypically and genotypically diverse.
- There is an urgent need for mechanistic studies focusing on host–*Klebsiella* interactions to determine why only some preterm infants develop infections caused by these bacteria. In addition, there is a need for *Klebsiella*–microbiota studies to elucidate the mechanisms contributing to establishment and maintenance of *Klebsiella* populations in the preterm infant gut microbiota.

## Open Access

Open access for this article was enabled by the participation of Nottingham Trent University in an all-inclusive *Read & Publish* agreement with Portland Press and the Biochemical Society under a transformative agreement with JISC.

## Abbreviations

AAHC: antibiotic-associated haemorrhagic colitis
BGC: biosynthetic gene cluster
EOS: early-onset sepsis
GA: gestational age
LBW: low birth weight
LOS: late-onset sepsis
NEC: necrotizing enterocolitis
NICU: neonatal intensive care unit
TM: tilimycin
TV: tilivalline
VLBW: very low birth weight
