# Supplementary material for: The role of Klebsiella populations in preterm infants
Source: Biochem Soc Trans. 2023 Apr 26;51(2):887–96. doi: 10.1042/BST20200325 (PMC10212511; doi:10.1042/BST20200325)
Supplement: Supplementary Material [file BST-51-887-s1.pdf]

**Supplementary Table 1.** Summary of recent retrospective/observational studies and case reports of preterm infants referencing *Klebsiella*

Abbreviations used in the table are defined in the footnote.

| Study information | Main findings | Reference |
|---|---|---|
| Investigation of neonatal outcomes of LOS patients in Oman (2007-2014); *n* = 125 LOS cases from 26,289 live births. | Most BSIs occurred in VLBW neonates (*n* = 81) and preterm infants (*n* = 74). Predominant organisms isolated were CONS (47.2 %), *Kle. pneumoniae* (16.8 %), *Esc. coli* (7.2 %), *Pse. aeruginosa* (5.6 %), *Bur. cepacia* (5.6%), *Enterococcus* spp. (3.2 %), MRSA (2.4 %), and *Ser. marcescens* (2.4 %). | [1] |
| Case report of premature neonate with signs of severe respiratory distress. | Spontaneous vaginal delivery at 30 weeks GA. Congenital brucellosis associated with *Kle. pneumoniae* co-infection. | [2] |
| Antimicrobial sensitivity pattern in neonatal sepsis in Bangladesh NICU (July-Dec 2017); *n* = 94. | 72.3 % premature neonates; 84.0 % LBW; 76.6 % EOS. 21.3 % of cases had positive blood culture, *Klebsiella* spp. were isolated in 35.0 % of positive cases. MDR *Kle. pneumoniae* and *Esc. coli* were leading causes of neonatal sepsis. | [3] |
| Investigation of bacterial endotracheal tube colonization in preterm infants in Brisbane (Australia) (2007- 2016); *n* = 1054 intubated preterm neonates (<32 weeks GA). | Three colonization groups: (1) no growth, *n* = 319; (2) normal respiratory bacteria only, *n* = 357; and (3) significant bacteria, *n*=378 – 40 % harboured Gram -ve bacteria and 34 % *Klebsiella* (which was most prevalent organism in the significant bacteria group).<br>Significant bacteria group had significantly lower birth weights (*P*<0.001) and younger GA (*P*<0.001) than no growth neonates.<br>The odds of septicaemia (10 % of cohort) were higher for normal respiratory bacteria group (2.6 times) and significant bacteria group (4.5 times) than for no growth group. Study did not determine causative agent of septicaemia. | [4] |
| Investigation of outcomes of neonatal sepsis patients in Bahawal Victoria Hospital (Pakistan) (Jan-June 2019); *n* = 586. | Neonatal sepsis based primarily on clinical features. 56 % preterm infants, and 51 % LBW. 415 EOS, 171 LOS. Positive blood cultures were seen in 8.9 % of patients; although microbiological analysis was not performed for 20.5 % of sepsis patients. Predominant organisms isolated were *Kle. pneumoniae* (36.5 %), *Esc. coli* (28.5 %), and *Sta. aureus* (15.4 %). 123 neonates died (21 %). | [5] |
| Bacterial colonization and primary sepsis at Hanover Medical School (Germany) (Nov 2016-Mar 2018); *n* = 584. | Gut and respiratory tract samples screened for MRSA, VRE and Gram -ve bacteria. 282 neonates were colonized with at least one target organism, 26.2 % of whom had MDR isolate(s). 534 bacterial isolates of target organisms were obtained, with the predominant organisms being *Esc. coli* (131 neonates), *Etb. cloacae* (106 neonates), *Kle. oxytoca* (72 neonates) and *Kle. pneumoniae* (49 neonates). 9 neonates were shown to have hospital-acquired *Kle. pneumoniae* (monoclonal cluster, over a 3-week period).<br>23 positive blood culture LOS cases; the predominant organisms isolated were *Staphylococcus* spp. (60.9 %), *Etb. cloacae* (8.7 %) and *Kle. aerogenes* (4.3 %). | [6] |
| *Klebsiella aerogenes* outbreak in Austrian NICU (Sept-Dec 2016); *n* = 16. | Sequencing confirmed outbreak of a yersiniabactin-producing *Kle. aerogenes* strain.<br>13 neonates were asymptomatic for *Kle. aerogenes* infection, some of whom developed LOS caused by another pathogen.<br>3 LBW preterm neonates developed NEC, 2 complicated by sepsis (1 of whom died). | [7] |

| Study information | Main findings | Reference |
|---|---|---|
| MDR *Enterobacteriaceae* in neonatal unit in Johannesburg (2013-2015); *n* = 2437. | 291 neonates with EOS (465 bacterial infections). Predominant organisms isolated were *Kle. pneumoniae* (66.2 %), *Etb. cloacae* (1.5 %), *Esc. coli* (9.6 %), *Ser. marcescens* (6.2 %) and other *Klebsiella* spp. (5.8 %). LBW and younger GA were associated with MDR EOS. *Ser. marcescens* EOS had highest mortality rate (55.2 % of mortalities). | [8] |
| Association of maternal UTI with neonatal UTI in Dumlupinar University NTU, Turkey (July 2017-Jan 2018); n = 230 singleton births. UTI classified as >$10^5$ CFU/ml of urine of one organism. | Statistically significantly higher rate of UTI (25.2 %) in study group (exposed *in utero* to maternal UTI) than control group (no maternal UTI) (7.8 %; *P*<0.001).<br>Most common organisms isolated from study group were *Esc. coli* (*n* = 15), *Klebsiella* spp. (*n* = 5), *Proteus* spp. (*n* =3) and *Serratia* spp. (*n* = 2). | [9] |
| Case report of *K. pneumoniae* acute chorioamnionitis. | *Kle. pneumoniae* acute chorioamnionitis caused intrauterine foetal death at 138 days GA (just under 20 weeks). *Kle. pneumoniae* isolated post mortem from foetal blood and lung samples. Maternal blood and urine samples taken after foetal death were culture negative. | [10] |
| Aetiology, clinical signs and laboratory parameters of acute bacterial meningitis in neonates at Ghaem hospital Mashhad NICU (Iran) (2009-2018); *n* = 468. | Meningitis confirmed by positive CSF culture and/or clinical signs. 233 had lumbar puncture with 36.5 % positive CSF culture. 94 % of confirmed meningitis cases were preterm infants (<37 weeks GA). Gestational disorders were seen in 55.3 % of meningitis neonates (PROM, 19 %; chorioamnionitis, 9 %; and hypertension, 7 %).<br>Positive blood culture was seen in 80 % of neonates with late-onset meningitis. *Kle. pneumoniae* (48.6 %) and *Kle. aerogenes* (14.3 %) were the leading causes of meningitis; with respiratory symptoms (94%) the most common clinical finding associated with meningitis. | [11] |
| Pathogen composition and clinical features of preterm infants with sepsis at Zhengzhou University NICU (China) (Jan 2014-May 2018); *n* = 371. | Sepsis confirmed by positive blood culture. 73 EOS (< 1 week old) and 298 LOS (≥ 1 week old). LOS group had significantly higher NEC rate (*P*<0.05). *Kle. pneumoniae* was the predominant bacterium isolated (27.4 % of EOS and 43.6 % of LOS cases), followed by *Esc. coli* (16.4 % EOS and 7.0 % LOS). | [12] |
| Case-control stool surveillance study investigating whether ESBL-producing *Enterobacteriales* (ESBL-E) are a risk factor for NEC in preterm infants (2005-2016); *n* = 217 ESBL-E cases, *n* = 217 matched controls. | 270 ESBL-E isolates from 217 preterm infants; 44 cases harboured more than one species. 10/217 ESBL-E cases developed NEC, compared to 2/217 matched controls. Predominant ESBL-E were *Kle. oxytoca* (46 %), *Kle. pneumoniae* (19 %), *Cit. freundii* (17 %). Faecal ESBL-E carriage was not a risk factor for NEC. | [13] |
| Bacterial epidemiology of neonatal sepsis in preterm infants in Ethiopia (July 2016-May 2018); *n* = 4,919 preterm neonates. | 2,003 presented with clinical signs of sepsis; 1,807 EOS, 196 LOS. Positive blood culture for 36.9 % of 690 blood samples tested. Predominant organisms isolated were *Klebsiella* spp. (33.9 %), CONS (18.2 %), *Sta. aureus* (16.9 %) and *Esc. coli* (5.2 %). 91.3 % of the blood isolates were MDR. | [14] |
| Bacterial epidemiology of culture-positive neonatal sepsis in South China (2012-2016); *n* = 597 cases. | 388 full-term infants and 206 preterm infants (11.9 % LBW; 9.0 % VLBW); no GA records for 3 neonates. 97 EOS, 502 LOS (some neonates experienced multiple episodes of sepsis caused by different pathogens). 620 isolates; *Kle. pneumoniae* (21.9%), *Esc. coli* (21.9 %), Group B *Streptococcus* (13.2 %), *Sta. aureus* (6.8 %). 57 sepsis patients died in hospital, with *Esc. coli* (*n* = 22) the leading cause of mortality, followed by *Kle. pneumoniae* (*n* = 12), Group B *Streptococcus* (*n* = 4), and *Sta. aureus* (*n* = 2). | [15] |

| Study information | Main findings | Reference |
|---|---|---|
| Association between tracheobronchial aspirate fluid isolates and BPD in VLBW preterm infants admitted to NICU (2015-2017); *n* = 155. | 41 neonates diagnosed with BPD. Significantly lower BW (*P*<0.001) and younger GA (*P*<0.01) for BPD group. Incidence of neonatal sepsis was higher in BPD patients. Positive tracheobronchial aspirate fluid cultures for 33/155 neonates; predominant organisms isolated were *Ste. maltophilia* and *Kle. pneumoniae*. Gram +ve cocci isolated from 14.6 % of BPD and 6.1 % of non-BPD patients, Gram -ve bacteria isolated from 39.0 % BPD and 8.8 % non-BPD patients. | [16] |
| Clinical characteristics and bacterial epidemiology of culture-positive neonatal sepsis in Guangzhou city (South China) (June 2011-June 2017); *n* = 1048. | Detailed clinical and microbiological data available for 297 cases. Predominant organisms isolated were *Esc. coli*, *Kle. pneumoniae* and CONS.<br>Full-term infants – *Esc. coli* (26 %), CONS 24%, *Str. agalactiae* (18 %); preterm infants – *Kle. pneumoniae* (31 %), *Esc. coli* (25 %), *Enterococcus* spp. (13 %). Caesarean section born infants – *Kle. pneumoniae* (30 %), *Esc. coli* (16 %), *Enterococcus* spp. (16 %), CONS (13 %); vaginally delivered infants – *Esc. coli* (32 %), *Kle. pneumoniae* (18 %), CONS (17%), *Str. agalactiae* (10 %). PROM cases – *Esc. coli* (35 %), *Kle. pneumoniae* (22 %), *Enterococcus* spp. (14 %). VLBW – *Kle. pneumoniae* (32 %), *Esc. coli* (20 %), *Enterococcus* spp. (14 %), *Candida* spp. (13 %); LBW – *Esc. coli* (36 %), *Kle. pneumoniae* (29 %), *Enterococcus* spp. (12 %); normal BW – *Esc. coli* (25 %), CONS (25 %), *Str. agalactiae* (15 %), *Kle. pneumoniae* (14 %). EOS – *Esc. coli* (32 %), *Str. agalactiae* (27 %), CONS (17 %); LOS – *Kle. pneumoniae* (29 %), *Esc. coli* (23 %), CONS (15 %). | [17] |
| Clinical and bacteriological profile of neonatal sepsis in Bhutan (2016); *n* = 314. | 232 clinically suspected EOS (24 blood culture positive), 82 clinically suspected LOS (20 blood culture positive). 48 bacteria isolated from 44 positive culture cases, with 64.6 % being Gram -ve bacteria. Predominant bacteria were CONS (31.0 %), *Kle. pneumoniae* (27.0 %) and *Acinetobacter* spp. (18.8 %). | [18] |
| Investigation of EOS among preterm neonates in China (April 2015-May 2018); *n* = 27,532 infants <34 weeks GA. | EOS confirmed by positive blood culture within 72 h of birth. 321 confirmed EOS cases, 61 of whom died within a week of EOS onset. Gram -ve bacteria were the predominant organisms isolated (61.7 %) and associated with 82.0 % of EOS deaths. *Esc. coli* (20.3 %) was the leading bacterium isolated, followed by CONS (16.5 %), *Ach. xylosoxidans* (9.0 %), *Kle. pneumoniae* (8.1 %), and Group B *Streptococcus* (2.5 %). | [19] |
| Investigation of sepsis and its association with neurodevelopmental outcomes in preterm infants (24-32 weeks GA) at NICU of Maternity hospital in Kuwait (2017); *n* = 203. | 16 EOS, 94 LOS. Higher cerebellar haemorrhage risk was seen with LOS. LOS group had lower BW, younger GA, and smaller length and head circumference that EOS and no sepsis groups. A significantly higher risk of cerebral palsy was seen in LOS group (28.0 %) compared to EOS (0.0 %) and no sepsis (9.6 %) groups (*P*<0.01). Significantly lower motor scores were seen for LOS group at 3 years of age, compared to EOS and no sepsis groups (*P*<0.05).<br>*Kle. pneumoniae* (31.3 %) most common organism isolated in EOS, followed by *Str. agalactiae* (25.0 %) and *Esc. coli* (18.8 %). *Kle. pneumoniae* (57.4 %) most common organism isolated in LOS, followed by other pathogens (27.7 % - including CONS, *Sta. epidermidis*, MRSA and *Serratia* spp.). | [20] |
| Case report of neonatal meningitis caused by *Kle. pneumoniae*. | Hypervirulent ESBL-producing *Kle. pneumoniae* strain causative agent of pyogenic meningitis in a full-term neonate. Isolated from blood and CSF samples. Despite antibiotic treatment (modified following confirmed *Kle. pneumoniae* culture), the patient died on 35$^{th}$ day of disease [32 days after hospitalization with suspected meningitis, fever (39 °C) and seizures]. | [21] |
| Investigation of epidemiology of culture positive EOS in Suzhou (China) (2011-2017); *n* = 306. | 128 full-term and 178 preterm infants. Predominant organisms isolated Group B *Streptococcus* [28.1 %; significantly higher in full-term (32.8 %) than preterm infants (18.3 %) (*P*<0.01)], *Esc. coli* (21.6 %), *Lis. monocytogenes* (11.8 %), and *Kle. pneumoniae* [7.8 %; significantly higher in preterm (10.2 %) than full-term infants (~3 %) (*P*<0.01)]. Bacteria were isolated from CSF of 30/220 who underwent lumbar puncture. | [22] |

| Study information | Main findings | Reference |
|---|---|---|
| | 30 patients had early onset meningitis, with the same bacteria cultured from both blood and CSF for 22 of them (the remaining 8 patients had negative blood cultures, but positive CSF cultures). | |
| Epidemiology of culture-positive sepsis among out-born patients in Northern India (Feb 2018-Jan 2019); *n* = 406. | 280 EOS, 126 LOS. Gram -ve bacteria (46.5 %), Gram +ve (27.6 %), yeasts (25.9 %). Predominant Gram -ve organisms isolated were *Kle. pneumoniae* (46.5%), *Aci. baumannii* (17.5 %), *Esc. coli* (8.0%), *Bur. cepacia* complex (7.4 %), *Aci. junii* (5.3 %), and *Ent. cloacae* (3.1 %). Gram -ve sepsis cases had higher rate of medical respiratory intervention (oxygen, mechanical) and lower survival rate (50.3 % vs 72.3 %: $P$<0.05) compared to Gram +ve sepsis cases. | [23] |
| Investigation of LOS in VLBW preterm infants in China (2012-2018); *n* = 710. | 171 LOS cases (24 %), 139 with positive blood culture (17 of whom also had CSF positive culture). Predominant organisms isolated were *Kle. pneumoniae* (32.4 %), *Sta. aureus* (11.7 %), *Aci. baumannii* (11.0 %), *Sta. epidermidis* (6.2 %). 30 LOS patients died, 25 of LOS, 3 of NEC, 1 of intraventricular haemorrhage and 1 of multiple organ failure. | [24] |
| Clinical characteristics, epidemiology and antimicrobial resistance associated with neonatal sepsis at Dhaka Medical College hospital NICU (Bangladesh) (Jan 2014-June 2015); *n* = 200. | 82 EOS, 118 LOS. LOS group had lower BW and younger GA than EOS group. Positive blood cultures were found in 55 % of sepsis cases, with *Kle. pneumoniae* the predominant bacterium isolated. | [25] |
| Investigation of suspected neonatal sepsis cases in Southern part of Nepal (Jan 2017-Feb 2018); *n* =1200. | 1024 clinically suspected EOS, 176 clinically suspected LOS. Positive blood cultures obtained for 28.32 % suspected EOS and 41.47 % suspected LOS cases. Predominant bacteria isolated for EOS cases were *Sta. aureus* (62.8 %), *Kle. pneumoniae* (14.1 %), *Streptococcus* spp. (7.9 %), *Pse. aeruginosa* (5.5 %), *Esc. coli* (4.8 %) and CONS (3.1 %). Predominant bacteria isolated for LOS cases were *Sta. aureus* (64.4 %), *Kle. pneumoniae* (9.6 %), *Streptococcus* spp. (6.8 %), *Pse. aeruginosa* (6.8 %), CONS (4.8 %) and *Esc. coli* (4.1 %). | [26] |
| Epidemiology and risk factors of culture positive childhood BSI at West China Second University Hospital (2016-2018); *n* = 228. | 174 cases (76.3 %) were caused by ESKAPE pathogens, 124 involving MDR bacteria. Predominant ESKAPE bacteria isolated were *Esc. coli* (26.8 %), *Kle. pneumoniae* (20.2 %), *Ent. faecium* (12.7 %), and *Sta. aureus* (12.7 %). Hospital stays were longer for patients with MDR ESKAPE BSI ($P$<0.01) and ESKAPE BSI ($P$<0.05), compared to non-ESKAPE BSI patients. MDR-ESKAPE BSI cases had more nosocomial infections ($P$<0.001) and underlying diseases ($P$<0.001) than all other BSI cases (50 non-MDR ESKAPE BSI and 54 non-ESKAPE BSI). | [27] |
| Surveillance of NI in a Bulgarian NICU (Jan 2017-June 2018); *n* = 507. | 54 NI recorded in 48 neonates. LBW and premature infants most at risk of NI. Predominant organisms isolated were *Kle. pneumoniae* (21.35 %), CONS (13.49 %), *Esc. coli* (11.24 %), *Pse. aeruginosa* (10.12 %), *Aci. baumannii* (10.12 %) and *Ent. faecalis* (7.86 %). | [28] |
| Perinatal infections in Ukraine (2017-2019); *n* = 18,348. | 472 perinatal infections recorded - including BSIs (35.6 %), lower respiratory tract infections (21.4 %), meningitis (18.2 %, conjunctivitis (11.9 %), skin and soft tissue infections (6.6 %), dacryocystitis (3.8 %) and UTI (2.5 %). LBW and younger GA were potential risk factors for neonatal infections, as well as PROM, maternal infection, respiratory distress during birthing, turbid and meconial amniotic fluid, and invasive procedures (e.g. intubation).<br>*Esc. coli*, *Str. agalactiae*, *Sta. aureus*, *Kle. pneumoniae*, CONS, *Ent. cloacae* and *Pse. aeruginosa* were the most common organisms isolated. | [29] |
| Investigation of prevalence and risk factors of antimicrobial resistant Gram -ve | 39 full-term and 80 preterm infants. 82 EOS, 37 LOS. *Kle. pneumoniae* was the predominant organism isolated (79 % cases), followed by *Esc. coli* (8 %). 11 sequence types (ST) of *Kle. pneumoniae* were identified. ST37 was more common among inborn, Caesarean-section-delivered LOS cases, implying hospital-acquired ST37 infections. | [30] |

| Study information | Main findings | Reference |
|---|---|---|
| bacteremia in Ethiopia (Mar-Dec 2017); *n* = 119. | | |
| Investigation of pathogenic bacteria associated with neonatal conjunctivitis in Chennai; *n* = 139. | Positive culture obtained from conjuncitivitis swabs in 92 cases.<br>CONS was predominant organism isolated (35.9 %), followed by *Kle. pneumoniae* (16.3 %), *Acinetobacter* spp. (16.3 %), *Sta. aureus* (14.1 %), *Pse. aeruginosa* (8.7 %) and *Esc. coli* (8.7 %). | [31] |
| Association of vaginal microbial dysbiosis with selected neonatal outcomes in Lodz (Poland) (Jan-June 2019); *n* = 809. | Vaginal swabs sampled on hospital admission for delivery. 48.95 % mothers had abundant aerobic bacteria and/or fungi (i.e. AVM). Significantly more preterm births in AVM group (9.09 %), than control group (5.31 %) ($P<0.05$) and significantly more perinatal infections in neonates from AVM group (23.97 %) compared to control group (15.94 %) ($P<0.01$). Most prevalent AVM vaginal bacteria isolated were *Str. agalactiae* (57.32 %), *Candida* spp. (39.64 %), *Klebsiella* spp. (9.85 %) and *Sta. aureus* (7.32 %). Higher incidence of signs of infection (35.90 % vs 19.16 %; $P<0.05$), and respiratory stress signs (18.00 % vs 6.28 %; $P<0.05$), in neonates whose mothers AVM included *Klebsiella* spp. | [32] |
| Clinical characteristics of EOS and LOS caused by *Kle. pneumoniae* (Children's Hospital of Chongqing Medical University, China) (2000-2019); *n* = 180. | 66 EOS, 114 LOS. 66 LOS patients had MDR *Kle. pneumoniae*, all other *Kle. pneumoniae* isolates were non-MDR. LOS patients had lower GA and LBW, higher rates of respiratory failure, BPD, and intraventricular haemorrhage, and lower rate of acute respiratory distress syndrome than EOS group ($P<0.05$). LBW, younger GA, longer antibiotic exposure, peripheral catheter insertion, mechanical ventilation and parenteral nutrition prior to sepsis diagnosis were more common in neonates with MDR *Kle. pneumoniae* sepsis than non-MDR *Kle. pneumoniae* group. | [33] |
| Investigation of EOS in VLBW infants in Qilu Hospital (Qingdao, China) (2014-2020); *n* = 69 VLBW infants. | 34 EOS, 14 LOS. Risk factors for EOS included contaminated amniotic fluid, intrauterine distress, PROM, and maternal fever.<br>Positive blood cultures (49 isolates) were obtained from the 48 sepsis cases. 27 Gram -ve bacteria [predominantly *Esc. coli* (*n* = 12; 8 EOS, 4 LOS) and *Kle. pneumoniae* (*n* = 9; 6 EOS, 3 LOS)], and 21 Gram +ve bacteria [predominantly *Sta. epidermidis* (*n* = 8; 6 EOS, 2 LOS) and *Str. agalactiae* (*n* = 7; all EOS)]. | [34] |
| Epidemiology of neonatal sepsis in East China (2016-2020); *n* = 707 neonatal BSI. | Preterm infants were more likely to be infected by Gram -ve bacteria. Predominant organisms isolated were CONS (67.4 %), *Esc. coli* (12.9 %), *Kle. pneumoniae* (9.1 %), *Str. agalactiae* (8.6 %) and *Sta. aureus* (3.3 %). Rates of *Esc. coli* and *Kle. pneumoniae* infections were similar in 2016 and 2017, but *Esc. coli* infections have significantly increased since then. | [35] |
| Epidemiology of ceftazidime/ avibactam-resistant CRKP in NICU (China) (July 2016-June 2017); *n* = 5348. | 43 CRKP isolates (10 of which were CZAR) from 300 *Kle. pneumoniae* isolates. Non-β-lactam antibiotic resistance spectrum of CZAR isolates was different to other CRKP isolates, with lower resistance rates to amikacin, levofloxacin, ciprofloxacin and nitrofurantoin. CZAR isolates had different carbapenemase genes to other CRKP isolates. All CZAR isolates had ESBL genes (with 9 carrying SHV-11) and CTX-M-14 gene. 8 different sequence types were identified from the 10 CZAR isolates. | [36] |

*Ach.*, *Achromobacter*; *Aci.*, *Acinetobacter*; AVM, abnormal vaginal microbiota; BPD, bronchopulmonary dysplasia; BSI, blood stream infections; *Bur.*, *Bulkholderia*; *Cit.*, *Citrobacter*; CONS, coagulase-negative *Staphylococcus*; CRKP, carbapenem-resistant *Klebsiella pneumoniae*; CSF, cerebrospinal fluid; CZAR, ceftazidime/avibactam-resistant; *Esc*., *Escherichia*; *Ent*., *Enterococcus*; *Etb*., *Enterobacter*; EOS, early-onset sepsis

(< 72 h after birth, unless otherwise stated); ESBL, extended-spectrum β-lactamase; GA, gestational age; Gram -ve, Gram-negative; LBW, low birth weight; *Lis.*, *Listeria*; LOS, late-onset sepsis (≥ 72 h old, unless otherwise stated); MDR, multidrug-resistant; MRSA, methicillin-resistant *Staphylococcus aureus*; NEC, necrotizing enterocolitis; NI, nosocomial infection; NICU, neonatal intensive care unit; PROM, premature rupture of membranes; *Pse.*, *Pseudomonas*; *Ser.*, *Serratia*; *Sta*., *Staphylococcus*; *Ste.*, *Stenotrophomonas*; *Str*., *Streptococcus*; UTI, urinary tract infection; VLBW, very low birth weight; VRE, vancomycin-resistant enterobacteria.

**REFERENCES**


1 Abdellatif, M., Al-Khabori, M., Rahman, A.U., Khan, A.A., Al-Farsi, A. and Ali, K. (2019) Outcome of late-onset neonatal sepsis at a tertiary hospital in Oman. *Oman Med. J.* **34**, 302–307 10.5001/omj.2019.60

2 Al-Faifi, J. and Ibrahim, M.E. (2022) Congenital brucellosis associated with subsequent *Klebsiella pneumoniae* co-infection in a premature neonate: A rare case report. *J. Infect. Public Health* **15**, 586–588 10.1016/j.jiph.2022.04.009

3 Amin, S.E., Hossain, M.A., Akhtaruzzaman, M., Choudhury, M.F., Islam, N., Hossain, C.F., et al. (2020) Antimicrobial sensitivity pattern in neonatal sepsis in neonatal intensive care unit of Mymensingh Medical College Hospital. *Mymensingh Med. J. MMJ* **29**, 784–792

4 Antoine, J., Inglis, G.D.T., Way, M., O'Rourke, P. and Davies, M.W. (2020) Bacterial colonisation of the endotracheal tube in ventilated very preterm neonates: a retrospective cohort study. *J. Paediatr. Child Health* **56**, 1607–1612 10.1111/jpc.15046

5 Atif, M., Zia, R., Malik, I., Ahmad, N. and Sarwar, S. (2021) Treatment outcomes, antibiotic use and its resistance pattern among neonatal sepsis patients attending Bahawal Victoria Hospital, Pakistan. *PloS One* **16**, e0244866 10.1371/journal.pone.0244866

6 Baier, C., Pirr, S., Ziesing, S., Ebadi, E., Hansen, G., Bohnhorst, B., et al. (2019) Prospective surveillance of bacterial colonization and primary sepsis: findings of a tertiary neonatal intensive and intermediate care unit. *J. Hosp. Infect.* **102**, 325–331 10.1016/j.jhin.2019.01.021

7 Baier-Grabner, S., Equiluz-Bruck, S., Endress, D., Blaschitz, M., Schubert, S., Indra, A., et al. (2022) A yersiniabactin-producing *Klebsiella aerogenes* strain causing an outbreak in an Austrian neonatal intensive care unit. *Pediatr. Infect. Dis. J.* 10.1097/INF.0000000000003553

8 Ballot, D.E., Bandini, R., Nana, T., Bosman, N., Thomas, T., Davies, V.A., et al. (2019) A review of multidrug-resistant *Enterobacteriaceae* in a neonatal unit in Johannesburg, South Africa. *BMC Pediatr.* **19**, 320 10.1186/s12887-019-1709-y

9 Bilgin, H., Yalinbas, E.E., Elifoglu, I. and Atlanoglu, S. (2021) Maternal urinary tract infection: is it associated with neonatal urinary tract infection? *J. Fam. Reprod. Health* **15**, 8–12 10.18502/jfrh.v15i1.6067

10 Bonasoni, M.P., Palicelli, A., Dalla Dea, G., Comitini, G., Nardini, P., Vizzini, L., et al. (2021) *Klebsiella pneumoniae* chorioamnionitis: an underrecognized cause of preterm premature rupture of membranes in the second trimester. *Microorganisms* **9**, E96 10.3390/microorganisms9010096

11 Boskabadi, H., Heidari, E. and Zakerihamidi, M. (2020) Etiology, clinical findings and laboratory parameters in neonates with acute bacterial meningitis. *Iran. J. Microbiol.* **12**, 89–97

12 Cheng, L., Xu, F.-L., Niu, M., Li, W.-L., Xia, L., Zhang, Y.-H., et al. (2019) Pathogens and clinical features of preterm infants with sepsis. *Zhongguo Dang Dai Er Ke Za Zhi Chin. J. Contemp. Pediatr.* **21**, 881–885

13 Eberhart, M., Grisold, A., Lavorato, M., Resch, E., Trobisch, A. and Resch, B. (2020) Extended-spectrum beta-lactamase (ESBL) producing *Enterobacterales* in stool surveillance cultures of preterm infants are no risk factor for necrotizing enterocolitis: a retrospective case-control study over 12 years. *Infection* **48**, 853–860 10.1007/s15010-020-01453-0

14 Eshetu, B., Gashaw, M., Solomon, S., Berhane, M., Molla, K., Abebe, T., et al. (2020) Bacterial isolates and resistance patterns in preterm infants with sepsis in selected hospitals in Ethiopia: a longitudinal observational study. *Glob. Pediatr. Health* **7**, 2333794X20953318 10.1177/2333794X20953318

15 Gao, K., Fu, J., Guan, X., Zhu, S., Zeng, L., Xu, X., et al. (2019) Incidence, Bacterial profiles, and antimicrobial resistance of culture-proven neonatal sepsis in south China. *Infect. Drug Resist.* **12**, 3797–3805 10.2147/IDR.S223597

16 Gao, X.-Y., Dai, Y.-H., Fan, D.-Z., Xie, X.-Y., Yang, G., Xiao, X., et al. (2020) The association between the microbes in the tracheobronchial aspirate fluid and bronchopulmonary dysplasia in preterm infants. *Pediatr. Neonatol.* **61**, 306–310 10.1016/j.pedneo.2019.12.010

17 Guo, J., Luo, Y., Wu, Y., Lai, W. and Mu, X. (2019) Clinical characteristic and pathogen spectrum of neonatal sepsis in Guangzhou city from June 2011 to June 2017. *Med. Sci. Monit. Int. Med. J. Exp. Clin. Res.* **25**, 2296–2304 10.12659/MSM.912375

18 Jatsho, J., Nishizawa, Y., Pelzom, D. and Sharma, R. (2020) Clinical and bacteriological profile of neonatal sepsis: a prospective hospital-based study. *Int. J. Pediatr.* **2020**, 1835945 10.1155/2020/1835945

19 Jiang, S., Hong, L., Gai, J., Shi, J., Yang, Y., Lee, S.K., et al. (2019) Early-onset sepsis among preterm neonates in China, 2015 to 2018. *Pediatr. Infect. Dis. J.* **38**, 1236–1241 10.1097/INF.0000000000002492

20 Kartam, M., Embaireeg, A., Albalool, S., Almesafer, A., Hammoud, M., Al-Hathal, M., et al. (2022) Late-onset sepsis in preterm neonates is associated with higher risks of cerebellar hemorrhage and lower motor scores at three years of age. *Oman Med. J.* **37**, e368 10.5001/omj.2022.41

21 Khaertynov, K.S., Anokhin, V.A., Davidyuk, Y.N., Nicolaeva, I.V., Khalioullina, S.V., Semyenova, D.R., et al. (2017) Case of meningitis in a neonate caused by an extended-spectrum-beta-lactamase-producing strain of hypervirulent *Klebsiella pneumoniae*. *Front. Microbiol.* **8**, 1576 10.3389/fmicb.2017.01576

22 Lu, L., Li, P., Pan, T. and Feng, X. (2020) Pathogens responsible for early-onset sepsis in Suzhou, China. *Jpn. J. Infect. Dis.* **73**, 148–152 10.7883/yoken.JJID.2019.243

23 Mahich, S., Angurana, S.K., Sundaram, V. and Gautam, V. (2021) Epidemiology, microbiological profile, and outcome of culture positive sepsis among outborn neonates at a tertiary hospital in Northern India. *J. Matern.-Fetal Neonatal Med. Off. J. Eur. Assoc. Perinat. Med. Fed. Asia Ocean. Perinat. Soc. Int. Soc. Perinat. Obstet.* 1–9 10.1080/14767058.2021.1939300

24 Ma, L., Peng, Q., Wang, W., Yang, Y., Chen, Y., Wang, L., et al. (2021) Late-onset sepsis in very low birth weight preterm infants: 7 years' experience at a tertiary hospital in China. *Pediatr. Neonatol.* **62**, 529–535 10.1016/j.pedneo.2021.05.011

25 Moni, S.C., Mollah, A.H., Banerjee, M., Khan, T.H., Sejuti, A. and Morshed, S.S. (2020) Neonatal sepsis: clinical characteristics, epidemiology and antibiotic sensitivity pattern of the bacterial pathogens in neonatal intensive care unit of a tertiary care hospital. *Mymensingh Med. J. MMJ* **29**, 366–375

26 Pandit, B.R. and Vyas, A. (2020) Clinical Symptoms, Pathogen Spectrum, Risk Factors and antibiogram of suspected neonatal sepsis cases in tertiary care hospital of southern part of Nepal: A Descriptive Cross-sectional Study. *JNMA J. Nepal Med. Assoc.* **58**, 976–982 10.31729/jnma.5094

27 Peng, X., Zhou, W., Zhu, Y. and Wan, C. (2021) Epidemiology, risk factors and outcomes of bloodstream infection caused by ESKAPEEc pathogens among hospitalized children. *BMC Pediatr.* **21**, 188 10.1186/s12887-021-02661-9

28 Rangelova, V., Raycheva, R., Kevorkyan, A., Krasteva, M. and Dermendzhiev, T. (2020) Surveillance of nosocomial infections in a Bulgarian neonatal intensive care unit. *Folia Med. (Plovdiv)* **62**, 753–761 10.3897/folmed.62.e50437

29 Salmanov, A.G., Ishchak, O.M., Dobarin, S.A., Susidko, O.M., Mosendz, O.V., Korniyenko, S.M., et al. (2021) Perinatal infections in Ukraine: results of a multicenter study. *Wiadomosci Lek. Wars. Pol. 1960* **74**, 2025–2032

30 Solomon, S., Akeju, O., Odumade, O.A., Ambachew, R., Gebreyohannes, Z., Van Wickle, K., et al. (2021) Prevalence and risk factors for antimicrobial resistance among newborns with gram-negative sepsis. *PloS One* **16**, e0255410 10.1371/journal.pone.0255410

31 Suhas, P., Vishnu, S. and Muthayya, M. (2021) Pathogenic bacteria and their antibiotic sensitivity in ophthalmia neonatorum. *Oman J. Ophthalmol.* **14**, 85–87 10.4103/ojo.ojo_22_21

32 Szubert, M., Weteska, M., Zgliczynska, J., Olszak, O., Zgliczynska, M., Kalinka, J., et al. (2021) The association between imbalances in vaginal microflora and duration of pregnancy as well as selected maternal and neonatal parameters. *Ginekol. Pol.* 10.5603/GP.a2021.0035

33 You, T., Zhang, H., Guo, L., Ling, K.R., Hu, X.Y. and Li, L.Q. (2020) Differences in clinical characteristics of early- and late-onset neonatal sepsis caused by *Klebsiella pneumoniae.* 10.1177/2058738420950586

34 Yu, Y., Huang, Q. and Liu, A. (2021) Analysis of pathogens, drug resistance, sensitive antibiotic treatment and risk factors of early-onset sepsis in very low birth weight infants. *Am. J. Transl. Res.* **13**, 12939–12948

35 Zhang, X., Li, Y., Tao, Y., Ding, Y., Shao, X. and Li, W. (2022) Epidemiology and drug resistance of neonatal bloodstream infection pathogens in East China Children's Medical Center from 2016 to 2020. *Front. Microbiol.* **13**, 820577 10.3389/fmicb.2022.820577

36 Zhou, J., Yang, J., Hu, F., Gao, K., Sun, J. and Yang, J. (2020) Clinical and molecular epidemiologic characteristics of ceftazidime/avibactam-resistant carbapenem-resistant *Klebsiella pneumoniae* in a neonatal intensive care unit in China. *Infect. Drug Resist.* **13**, 2571–2578 10.2147/IDR.S256922
